# Reducing the Number of Mismatches between Hairs and Buccal References When Analysing mtDNA Heteroplasmic Variation by Massively Parallel Sequencing

**DOI:** 10.3390/genes11111355

**Published:** 2020-11-16

**Authors:** Kristiaan J. van der Gaag, Stijn Desmyter, Sophie Smit, Lourdes Prieto, Titia Sijen

**Affiliations:** 1 Netherlands Forensic Institute, Division of Biological Traces, Laan van Ypenburg 6, 2497GB The Hague, The Netherlands; s.smit@nfi.nl (S.S.); t.sijen@nfi.nl (T.S.); 2 NICC—Belgian Institute for Forensic Science and Criminology, Vilvoordsesteenweg 100, B-1120 Brussels, Belgium; Stijn.Desmyter@just.fgov.be; 3 Grupo de Medicina Xenómica, Instituto de Ciencias Forenses, Universidade de Santiago de Compostela, 15705 Santiago de Compostela, Spain; lourditasmt@gmail.com; 4 Laboratorio AND, Comisaría General de Policía Científica, 28039 Madrid, Spain; 5 Swammerdam Institute for Life Sciences, University of Amsterdam, Science Park 904, 1098XH Amsterdam, The Netherlands

**Keywords:** MPS, control region, heteroplasmy, mitochondrial, Sanger, detection threshold, Miseq, sequencing

## Abstract

In forensics, mitochondrial DNA (mtDNA) analysis is foremost applied to rootless hairs often lacking detectable nuclear DNA. Sanger sequencing is the routine mtDNA method in most forensic laboratories, even though interpretation of mixed samples and heteroplasmic sites can be challenging. Individuals may hold cells with low-level heteroplasmy variants below the detection threshold and other cells where this minor variant is the major one. This difference may be interpreted as a mismatch between reference and evidentiary trace samples, such as buccal specimens and rootless hairs. Such mismatches may be solved by Massively Parallel Sequencing (MPS), allowing more sensitive quantitative analysis for mixed positions than Sanger. The mtDNA control region was analysed in buccal reference samples from 26 individuals and 475 corresponding hairs by MPS and compared to Sanger sequencing data generated on the same samples. With MPS, mixed contributions down to 3% were regarded, leading to a substantial increase in the frequency of heteroplasmy. Our results demonstrate that previously reported mismatches between buccal reference and hair shaft samples by Sanger are detected as low-level heteroplasmy by MPS. A detailed overview of buccal and hair heteroplasmy is provided and implications for MPS-based mtDNA analysis in the context of forensic cases are discussed.

## 1. Introduction

**Figure 1:**
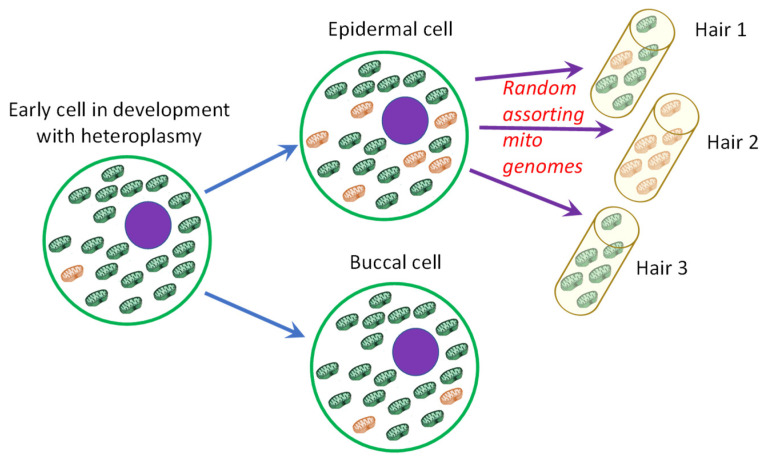
Illustration of shifts in heteroplasmy (HP) levels. Levels of HP can change during development. Especially for hairs, random assorting of mitochondrial genomes can lead to large shifts in HP levels. Once the HP level in the buccal cells is below the detection threshold of the analysis method, this may lead to an (apparent) homoplasmic mismatch with hair 2.

Since nuclear DNA is often very limited or absent in DNA samples derived from rootless hairs [1], analysis of mitochondrial DNA (mtDNA) is common practice for hairs recovered from a crime scene or used in identification cases. In most forensic laboratories, Sanger sequencing is the routine method for this mitochondrial analysis. The interpretation of mtDNA Sanger results is well described and standardised by international guidelines [2] and thereby usually relatively straightforward. However, mtDNA interpretation becomes more challenging when samples are mixed or exhibit heteroplasmy (HP). HP is common for mtDNA due to its high mutation rate [3]. However, due to mtDNA bottlenecks, different levels of HP can be observed within an individual which is especially apparent for hairs [4,5]. Intra-individual variation in the level of heteroplasmy may occur as replicated mtDNA genomes carrying mutations are randomly assorted into daughter cells. It was shown before that the HP levels of hairs are more related to those of buccals than those of blood cells [6], which is convenient in the forensic context where foremost buccal cells are taken as reference material. Still, in extreme cases, a mismatch between a hair and the corresponding buccal reference samples may occur when a hair appears homoplasmic [7], as illustrated in Figure 1.

The level of HP that is detected depends on the sequencing technique that is used and the analysis thresholds that are used for data analysis. With Sanger sequencing, secondary sequence peaks are generally called when they exceed a height of 10–20% of the primary peak, while the exact threshold can vary depending on the DNA position and used software (settings) [4,8]. Massively Parallel Sequencing (MPS) enables more sensitive analysis of mixed positions and could provide HP analysis down to the microheteroplasmy level (i.e., mutation for 2–5% of the mtDNA molecules) [9]. The different scenarios for comparing data from methods using different analysis thresholds are illustrated in Supplementary Figure S1. To gain insight into the variation in HP in rootless hairs at the sensitivity level of MPS-based analysis, we analysed 475 hairs and 26 buccals from corresponding individuals for the mtDNA Control Region. The samples were previously analysed by Sanger sequencing (part of the Sanger data is described in [4,7]). The first study analysed the complete control region (16,024–576). The second study focused specifically on point heteroplasmy (PHP) variation at mtDNA positions 16,093 or 16,182 and 16,183. The influence of using an expanded (3%) detection level for MPS data on the interpretation of hairs in forensic casework is discussed. Further, the observed variations for sites located within, or adjacent to, C-stretches are discussed focusing mostly on PHP and to a lesser extent on length heteroplasmy (LHP).

## 2. Materials and Methods

### 2.1. Sample Collection, DNA Extraction

Head hairs and buccal samples were collected from staff members of the NICC and the Spanish Forensic Police (CGPC) after signing informed consent (according to NICC regulations) for a total of 26 European individuals. Cleaning of hairs and DNA extraction was performed at the NICC for 25–50 hairs per individual. After removing the roots and a 0.5–1 cm proximal fragment of the hair, the first proximal 2 cm of the remaining hairs was used for extraction, as described previously [4]. Reference and hair samples were extracted separately. Both types of DNA extractions (references and hairs) were run in parallel with negative controls.

### 2.2. Sanger Sequencing

Sanger sequencing was performed by the NICC as described previously [4,7]. Hair samples P1–P6 and P8–P11 were analysed for the complete Control Region [4], while the remainder of the hair samples was analysed for positions 16,182 and 16,183 or 16,093 only [7]. Analysed ranges and haplotypes detected in the buccal cells of the 26 donors by Sanger [4,7] and MPS are shown in Supplementary Table S1.

### 2.3. mtDNA Quantification and MPS Sample Selection

For hair samples, DNA extracts of the previous studies were used for MPS preparation, while for buccal reference samples, new extracts of the reference samples were used. DNA quantification was performed by a multiplex qPCR amplifying an ALU-repeat and DYZ5 fragment as described by Nicklas and Buel [10], plus two mtDNA fragments with a length of 217 and 70 bp (protocol available upon request) using an AB7500 Real Time PCR System (ThermoFisher, Waltham, MA, USA). The number of mtDNA copies used as input for the Control Region amplifications was deduced from the 217 bp fragment. Based on the measured DNA concentration and the volume of DNA extract remaining, per individual 14 to 20 hair extracts were selected.

### 2.4. MPS Sequencing Preparation

The complete Control Region was amplified by ten fragments in two multiplex PCRs using an adjusted version of the mitominis protocol [11] updated for Miseq Sequencing [12]. All PCRs were performed on a Biometra Tadvanced thermocycler (Analytik Jena AG, Jena, Germany) using Qiagen Multiplex PCR mix (Qiagen, Hilden, Germany), 0.021–0.24 µM of the various primers [12] and a 35 cycle PCR of 20 s at 94 °C, 20 s at 55 °C and 20 s at 72 °C. Where possible, 500 copies of mtDNA were used in the PCR.

For library preparation, 1.5 µL of the (unpurified) amplified fragments for each multiplex were used in the KAPA Hyper Prep Kit (Roche, Basel, Switzerland) according to the manufacturer’s protocol with the exception that half reaction volumes were used for each step and that the final adapter concentration was 0.2 μM. Libraries were purified using Ampure XP beads (Beckman, Brea, CA, USA) with a 0.6 bead-to-volume ratio and elution in 40 µL water. Libraries were quantified using the KAPA SYBR^®^ FAST qPCR kit as described previously [13].

Sequencing was performed on the MiSeq FGx (Verogen, San Diego, CA, USA) using v3 chemistry for a minimum read length of 230 bp for each paired-end read; 88–96 samples were pooled in each sequencing run.

### 2.5. MPS Data Analysis

Paired-end reads were combined using an adjusted version of FLASH as described previously [13,14,15]. Data analysis was performed using FDSTools with an allele calling threshold of 3% with respect to the highest allele of a marker (fdstools—m 3) and a minimum allelic read count of 30 reads after noise correction (based on a training set including samples with different lengths of C-stretches [12]). Each sample was checked for fragments containing a minor mixed contribution (>3%) with a haplotype that differed for more than one site from the major haplotype indicating contamination. In addition, for samples containing three or more mixed sites, minor haplotypes were compared to all other samples in the same PCR or sequencing run as well as to the analyst’s haplotypes. Samples with suspected contamination were discarded from further analyses.

For this study, any HP site (PHP as well as LHP) that exceeds this threshold is considered heteroplasmic (and referred to as HP in the following paragraphs).

## 3. Results

The possibility to type HP at a much lower level by MPS impacts the forensic comparison of evidentiary samples and references (as illustrated in Supplementary Figure S1). The highly sensitive and truly quantitative information that is obtained by MPS provides detailed insight into HP variation within a person arising from the mtDNA bottleneck phenomenon [5]. This improved sensitivity can be specifically informative for forensic comparisons of reference and hair specimens.

In the discussion, we have excluded LHP in HV2 and HV3, and any discussed LHP in this study is therefore limited to the HV1 C-stretch. Sites located in the middle of a potential C-stretch (T16189, T310 and T318–T319, surrounded by Cs at both sides) are regarded separately in the discussion as these sites show divergent patterns of variation even when the C-stretch is interrupted (further referred to as “PHP C-stretch-related sites”). Interpretation of these “PHP C-stretch-related sites” is different between MPS and Sanger as every MPS read represents a single molecule rather than the consensus signal of Sanger where LHP variation adjacent to the sites overlaps with the signal of the site itself.

### 3.1. HP Occurrence in Buccal Reference Samples Analysed with MPS

All 26 buccal reference samples were analysed through MPS. All mixed positions were identified, and the HP levels were categorised (percentage contribution of the minor variant), as shown in Table 1. At lower levels, HP occurs more frequently. On average, a total of 1.8 PHP events per sample were observed by MPS analysis and 0.15 PHP events per sample by Sanger (haplotypes typed by Sanger and MPS are displayed in Supplementary Table S1). Six buccal samples were only typed for HV1 and HV2, but none of the observed HP sites in these samples by MPS were located outside of the HV1 and HV2 region. In addition, LHP in HV1 was observed for half of the buccal samples showing 16189C when the transition resulted in an uninterrupted homopolymer of nine or more C residues. Many low-level PHP events (48% of the total number of HP events) were located in or around the HV2 C-stretch (position 310, 316, 318 and 319) or at HV1 position 16,189.

From the MPS-typed PHP events, most events with levels > 20% were originally typed by Sanger as expected (scenario 1 of Supplementary Figure S1), except for 16320Y in X1. While 16320Y reached a level of 46% in the MPS analysis of this sample, a new inspection of the Sanger profile revealed only a minimal signal of the T-variant in the Sanger sequence (Supplementary Figure S2). From the sites in the 10–20% MPS category, one out of four events was also typed by Sanger (the remaining MPS-typed HP sites follow scenario 2 of Supplementary Figure S2).

It should be noted that new buccal swabs were used for the MPS analysis with sampling moments of up to several years between the Sanger and MPS samples which could explain some variation between the Sanger and MPS results. HP has been shown to accumulate with age [16]. However, since most low-level buccal HP sites are also observed in at least part of the corresponding hairs (taken around the time of the Sanger buccal sample), the observed difference is more likely to be caused by the difference in the detection level than by the relatively small age difference of the individuals at the sampling moments of the buccal references.

### 3.2. MPS Analysis of Buccal References Resolves Mismatches between Buccals and Corresponding Hairs Seen with Sanger Sequencing

**Figure 2:**
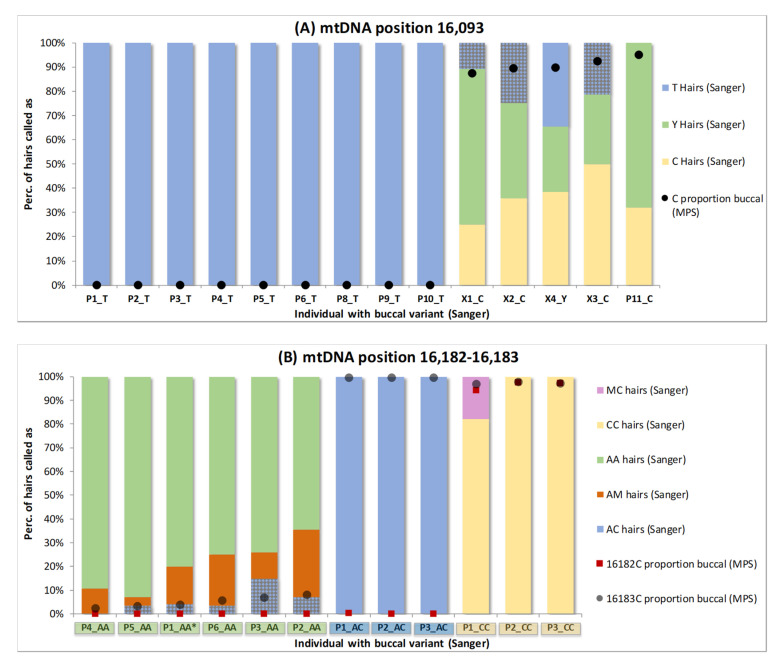
Overview of the percentage of hairs typed as specific genotypes (by Sanger) and the Sanger and Massively Parallel Sequencing results for the corresponding buccal reference. Two locations are regarded: position 16,093 (**A**) and positions 16,182 and 16,183 as a group (**B**). The MPS buccal levels of 16093C, 16182C and 16183C are marked as dots. For each individual, the Sanger call for the buccal reference sample is displayed after the sample name with a colour coding alike the hair. Sample naming is according to Supplementary Table S1 and [4,7] (* except for P1_AA which is the same sample as P5 in (**A**)) and involves 25 different persons: 14 in (**A**) and 12 in (**B**). The bars representing hairs that exhibit a mismatch with the respective buccal (by Sanger) are filled with a pattern (grey blocks).

The increased sensitivity of mixed position detection by MPS revealed more HP events in the buccal reference samples. This may reduce the number of apparent homoplasmic mismatches when buccal references are compared to individual hairs. To focus the analyses, we specifically regard the two variable locations for which the samples had been selected and studied by Sanger sequencing (including 11 of the 18 “PHP events at other sites” from Table 1): position 16,093 (14 samples), and positions 16,182 and 16,183 as a group (12 samples) [7]. Sanger-sequenced data of the hairs [4,7] were compared to both Sanger- and MPS-analysed data of the corresponding buccal references (Figure 2).

When regarding position 16,093, only one of the 14 buccal references showed a C/T (Y) PHP upon Sanger sequencing (X4, Figure 2A), while for four references, only a C was detected, and for nine references, only a T was detected. Notwithstanding, not only the Y-typed reference, but also all four C-typed references showed different genotypes for the Sanger-sequenced hairs: X1, X2, X3 and X4 showed T, Y and C hairs and P11 showed Y and C hairs. The Sanger T-typing for X1, X2 and X3 (in total 16/54 hairs) is a mismatch with the C-typed reference (Figure 2A, patterned bars). When the MPS data for the buccal references are regarded (with an analysis threshold of 3%), not only X4 but also X1, X2, X3 and P11 are typed as Y (black dots Figure 2A, following scenario B of Supplementary Figure S1) and these mismatches become matches. When regarding location 16,182–16,183, mismatches between Sanger-sequenced hairs and buccals are seen for P1_AA (P5 in [4,7]), P2_AA, P3_AA, P5_AA and P6_AA (in total 9/164 hairs) at position 16,183 (A-typed in buccals, C-typed in hairs). Again, these mismatches are resolved when the MPS buccal results are used as low-level HP is detected (patterned bars Figure 2B, following scenario B of Supplementary Figure S1).

Overall, due to its increased sensitivity, MPS indicated HP in the buccal references of 12 of the 26 individuals (P4_AA, P2_CC and P3_CC also show a minimal mixed contribution on position 16,183, but not within the MPS detection limit (scenario C of Supplementary Figure S1)), while Sanger indicated HP for only one individual. When the Sanger reference data are used, for eight individuals, mismatches appear with homoplasmic hairs (25 events in total), but these mismatches are resolved when the MPS reference data are used as the homoplasmic hair variant corresponds to the minor heteroplasmic variant in the buccal reference. Thus, it is unlikely that these apparent homoplastic hair variants represent de novo mutations.

For other HP positions, the overall HP level variation is further discussed in Section 3.3 and Section 3.4.

### 3.3. Minor Buccal HP Variants Observed as Apparent Homoplasmy or High-Level HP in Hairs by MPS

**Figure 3:**
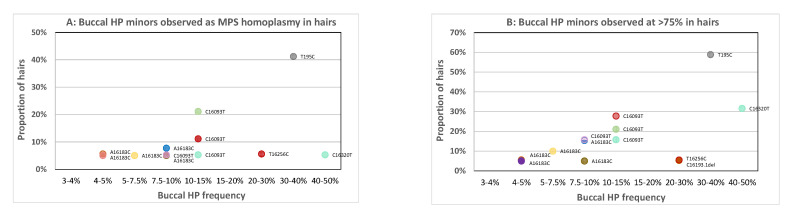
Proportion of hairs for which the minor HP variant of the corresponding buccal was observed as (**A**) an MPS homoplasmic variant or (**B**) approached homoplasmy (with a contribution of >75%). Different colours represent HP sites from different individuals. Sites that did not reach MPS homoplasmy or 75% are not plotted since the majority of dots would overlap. The sites involved are displayed next to the dot depicted as the buccal major nt followed by the position and the major nt observed in the plotted hairs.

In the examination of three PHP sites in the previous section, we observed that the minor variant of a PHP site in the buccal can occur as an apparent homoplasmy in a portion of the hairs by Sanger (Figure 2A: T-typed hairs for X1, X2, X3 and X4; Figure 2B: AC-typed hairs for P1_AA (P5 [4,7]), P2_AA, P3_AA, P5_AA and P6_AA). Next, the overall occurrence of the phenomenon was examined by considering all HP events in the Control Region for all 26 individuals. Since MPS is more sensitive in detecting HP than Sanger sequencing, MPS analysis was used for both the 26 buccal references and the 475 corresponding hairs. We consider MPS homoplasmy when no minor variant exceeding the 3% allele calling threshold is observed (which does not exclude low-level heteroplasmy below 3%). Figure 3A and Supplementary Table S2 display the proportion of hairs with MPS homoplasmy of the minor buccal HP variant categorised by the corresponding level of HP in the buccal. Narrow ranges were used for low-level buccal HP categories and broader ranges for higher-level categories. As the lower categories are closest to the detection limit, they are important to gain insight down to which buccal HP level the minor HP variant can reach homoplasmy in corresponding hairs.

There are 11 apparent MPS homoplasmic occurrences of the minor buccal HP variant in the hairs (Figure 3A), all involving PHP; five different HP sites are involved (195, 16,093, 16,183, 16,256, 16,320) and the frequency of the minor variant in the buccal reference is >4.5%. We also regarded the proportion of hairs approaching (>75% contribution of the buccal minor) homoplasmy, which roughly resembles hairs that could appear homoplasmic upon Sanger sequencing (Figure 3B). Now, 13 incidents are observed including one additional LHP position (16193del), all with an HP level in the buccal above 4%. Sites 16,093 and 16,183 are most frequently involved since most individuals were selected for PHP at these positions [4,7]. This finding confirms the mismatch results found with Sanger sequencing of hairs as shown in Figure 2 for sites 16,093 and 16,183. For site 16,183, it is noticeable that the buccal PHP levels are relatively low compared to other sites in the buccal references (between 4 and 10% only) even though occasionally high levels in hairs are observed. For all individuals involved, 16183C is directly adjacent to a long C-stretch (≥10 Cs) that occurs from a predominant C polymorphism at 16,189. Since, also for MPS, C-stretches and adjacent positions tend to exhibit increased error rates [9], it could be that the obtained levels of the C-variant for 16,183 in the buccal samples are somewhat biased (here causing either an underrepresentation of the 16183C level in buccals or an overrepresentation of 16183C homoplasmy in hairs).

It is important to note that no MPS homoplasmic hairs were seen for which the variant was not detected as a (low-level) HP in the buccal reference. Interestingly, also the 16320Y HP that was hardly visible in the Sanger reference sample (Supplementary Figure S2) was observed homoplasmic in one of the corresponding hairs and with >75% contribution in 32% of the hairs. Except for the previously discussed cases, no other Sanger buccal-to-hair mismatches were observed. In this study, we analysed 26 individuals and 475 corresponding hairs. While the numbers of hairs are substantial, the number of individuals is not sufficient to conclude that a complete mismatch between a buccal reference and a hair of the same individual cannot occur with MPS at all. For non-C-stretch-related positions, the buccal reference HP levels were at least 7.5%. Since this exceeds the 3% MPS analysis threshold by more than 2-fold, it is not very likely to observe an apparent MPS homoplasmic mismatch for a buccal and hair within the same individual. This is further supported by the fact that none of the HP minor variants with levels <4% were observed at an HP level of >75% in any of the tested hairs. However, since this study includes a total of 26 different haplotypes, it cannot be excluded that specific haplotypes or variants exist with a different pattern of HP variation.

Interestingly, in the buccal reference samples, all individuals carrying a C for 16,093 seem to have some level of PHP, while all individuals carrying a T appear to be homoplasmic, also at MPS resolution. This confirms the observations obtained with Sanger sequencing and indicates that 16093T is less prone to mutation than 16093C, as suggested before in several studies [3,7,17].

### 3.4. Variation between the Observed HP Frequencies in Buccals and Corresponding Hairs by MPS

**Figure 4:**
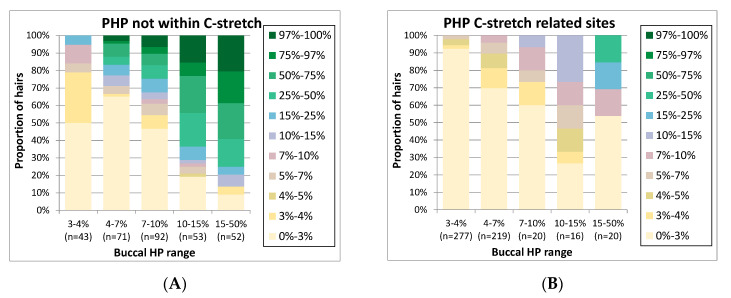
For point heteroplasmy (PHP) events observed in buccal references (grouped in PHP level categories on the *x*-axis), the proportion of hairs in various PHP level categories (see colour coding) is displayed (total number of investigated occurrences in hairs is indicated as n on the *x*-axis). The figure is divided in (**A**). Data for positions located within, or directly adjacent to, C-stretches are displayed separately (**B**) from non-C-stretch-related positions (**A**).

When a PHP site is detected in both a trace and a reference sample, it can provide additional confirmation that both samples may derive from the same individual. It is informative to assess how often PHP variants with a specific level in reference buccals are reproduced in hairs and vice versa. To examine the overall relation between the PHP levels in buccal references and corresponding hairs, Figure 4 was generated. As PHP sites located within potential C-stretches exhibited a different pattern of PHP variation, we analysed these as separate groups (Figure 4A,B). On the *y*-axis, the proportion of hairs is presented categorised by the hair PHP level, while on the *x*-axis, categories of PHP levels for the buccal samples are displayed. In the 26 buccal samples, HP was observed at 16 different positions: 1 LHP (position 16,193), 5 C-stretch-related and 10 non-C-stretch-related sites. The total number of buccal HP occurrences in the 26 individuals was 60 for buccals; the total number of investigated occurrences for these positions in hairs was 1099 (236 LHP, 552 C-stretch-related PHP and 311 non C-stretch-related PHP).

In general, Figure 4 shows the trend that with higher PHP levels in buccals, high PHP levels in hairs are seen more frequently. However, the distribution of PHP levels in hairs is broad, as is expected from mtDNA bottleneck assorting during hair development [5]. For instance, for buccal sites with a PHP level above 15%, >90% of the hairs show the PHP as well (categories 3–97%, Figure 4A), at levels in a very wide range. For buccal sites with PHP levels of 7–10%, PHP is absent in a much larger percentage of the hairs (47% of the hairs in category 0–3%, Figure 4A). Buccal PHP sites with levels below 10% are generally absent in the majority of hairs. Therefore, it could be considered to record PHP variants in databases only if levels exceed 10% and compare the lower-level PHP variants only in the case of a single mismatch. As this percentage approaches the PHP levels observed by Sanger sequencing, this strategy would also avoid large differences between database entries generated by Sanger or MPS.

Interestingly, mixed sites located on positions that lead to C-stretches tend to concur with lower mixed levels in hairs compared to other PHP sites while the extracts and libraries were processed in the same way. Since the C-stretch-related PHP sites were generally low-level, this might partly be the result of bias introduced by C-stretch-related sequencing errors seemingly causing an underestimation of HP levels of the C-stretch variant. For example, if we focus on position 310 (where a T > C SNP leads to a long C-stretch) as shown in Supplementary Figure S3, PHP is frequently observed at low levels. A trend is observed of rising frequencies in the hairs for individuals with a higher frequency in the buccal, but the frequencies in the hairs are much lower than those observed for other PHP sites (Figure 4A). While a portion of the seemingly PHP in the buccals could originate from PCR or sequence artefacts, reads of the molecules containing a long C-stretch are more likely to fail quality criteria during the basecalling process, resulting in biased lower levels of the C-stretch variant. Thereby, they will more often fall in the category below the allele calling threshold. However, the same errors would be expected in hairs and buccals, so the exact cause of the difference between these two remains unclear. For LHP sites, no clear trend was observed in the levels between buccals and hairs (Supplementary Figure S3C).

### 3.5. Mixed Sites Observed in Hairs, but Not in Corresponding Buccals

Up to now, we examined the concurrence of buccal HP sites in hairs. Besides, 36% of the hairs were found to carry mixed positions (above the 3% calling threshold) on sites where no HP was observed in the corresponding buccal reference sample. A total of 579 mixed occurrences were observed in the 475 analysed hairs. These were dispersed over 162 different positions, and for 70 positions, the same mixed position was seen in more than one hair. A total of 306 instances (44 positions) involved at least two hairs of the same individual, suggesting that this HP might exist throughout the cells of these individuals but below the MPS detection level in buccals. The mixture levels were mostly very low (273 of the 579 occasions had a mixture level between 3 and 5%; 93 resided above 10%). Further, multiple “non-buccal” mixed sites could be found in the same hair, but none of the hairs showed more than one mixed site exceeding 10%.

While most hairs exhibit a maximum of three mixed HP sites, 12 hairs stood out since they contained 4–8 mixed sites at low levels. Interestingly, 11 of these 12 hairs belong to the same individual and the mixed sites were all in the same fragment that contains a C-stretch of 10 Cs (due to an insertion of four Cs after position 573). Since all the mixed sites in this fragment represent additional Cs (although mostly not adjacent to the C-stretch itself, Supplementary Figure S4) and they are observed at similar levels in the buccal (although some just below the detection threshold), it suggests that this specific sequence results is an accumulation of errors rather than being actual mixed sites (the sites were excluded for other calculations in this paper). The suspected errors are present in the raw sequencing data from the instrument, so they could reside from either the PCR, the sequencing process or from the basecalling process in the sequencer.

**Figure 5:**
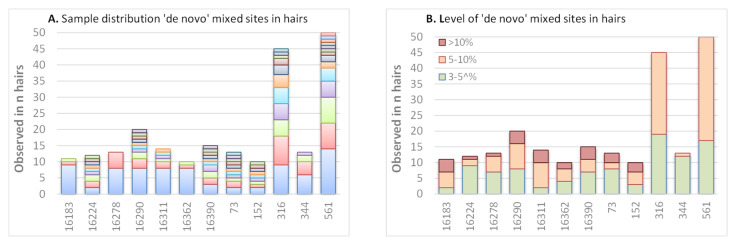
Top positions (observed in ≥10 hairs) for which mixed sites were observed in hairs but not in the corresponding buccal reference. (**A**) Distribution over different individuals, ordered by most frequent individual at the bottom to least frequent individual at the top (samples ordered per site by frequency: the same sample can have a different colour for different sites). (**B**) Distribution for various categories of mixed level.

Since several samples were specifically selected for containing a PHP at mtDNA positions 16,093, 16,182 or 16,183 [4,7], the tested samples do not represent a random population and seemingly “de novo” HP events in hairs could only be studied for positions that were not already HP in the buccal of the sample. From these sites, 12 HP sites stand out since they are observed in >10 hairs; all were observed in at least two hairs of one individual (Figure 5A). Some positions are limited to two or three individuals (A16183M, and C16278Y); others are common and seen in at least eight individuals (T16224Y, C16290Y, G16390R, A73R, T152Y, G316R and A561M). Although these 12 HP sites shown in Figure 5 stand out for the frequency at which HP is seen among hairs, the level of HP does not specifically stand out (Figure 5B); of the 93 positions that have a mixture rate of >10%, only 26 are at these 12 HP sites. Although these sites stand out for the frequency at which HP is seen among hairs, the level of HP does not specifically stand out (Figure 5B); of the 93 events with a mixture rate of >10%, only 26 are at these 12 HP sites.

In general, heteroplasmy detection is more sensitive with MPS than with Sanger sequencing, but its interpretation depends on several factors, such as background noise, coverage and strand bias. The authenticity of heteroplasmy also depends on the contamination rate; several examples can be found in the literature [18,19] where some of the reported heteroplasmies are most likely the result of contamination [8].

Although contamination cannot be totally ruled out, three of these variants (C16290Y, G16390R and A561M) were not present in any of the samples that were prepared together with the hairs, nor in any of the positive and negative controls or the haplotypes of the analysts. Since most of the haplotypes differ for two or more positions from each other, contamination would mostly result in multiple (linked) mixed sites, which was not the case. While the number of individuals is too low to look at potential haplogroup-specific patterns of HP variation, data from the hairs suggest that these seven common “de novo HP sites” might be more prone to HP formation than other positions. Interestingly, position 152 overlaps with the five most frequent PHP sites in buccal cells and blood samples, as reported by Irwin et al. [3] with Sanger sequencing. PHP at positions 16,183, 16,224, 16,278, 16,290, 16,311, 16,362, 16,390, 73 and 152 were previously found in [20] or in [3] or in both, while 316, 344 and 561 were not. From these three positions for which PHP has not been observed previously, 316 and 561 are adjacent to repeated Cs, suggesting that sequencing errors might be a factor here which would explain why they were observed mixed more frequently than other positions.

## 4. Discussion

Decreasing the mtDNA HP detection threshold to 3% upon applying MPS shows that previously observed mismatches between buccal references and corresponding hairs by Sanger sequencing are now explained as low-level HP that is present in the buccal reference. However, de novo mutations may occur as well, as illustrated by mixed positions seen in hairs and not in buccal references, though not observed in levels leading to homoplasmic MPS mismatches.

While the interpretation of buccal–hair comparisons becomes less complex as homoplasmic mismatches within the same individual are less likely to occur by using a lower detection threshold, the increased number of HP events adds a new level of complexity to the interpretation. It is therefore important to provide insight into the prevalence of low-level buccal HP variants as the HP site in hairs and vice versa.

For recording references in a database, it could be considered to only record HP variants with levels > 10% in buccal cell samples as lower-level variants will be less informative since they are often absent in hairs and are not likely to concur with a homoplasmic event of the minor in hairs. Low-level HP variants could, however, help to interpret comparisons of references and hairs (or other tissues) in case only a single mismatch is observed.

It should be noted that the results in this study are obtained by MiSeq sequencing of the used PCR-based assay for the mtDNA Control Region. The use of a different assay or sequencing platform may result in a different dynamic of HP reproducibility.

## 5. Conclusions

To gain insight into the variation in HP in rootless hairs at the 3% sensitivity level of MPS-based analysis, we analysed 475 hairs and 26 buccals from corresponding individuals for the mtDNA Control Region.

HP in buccals can concur with a broad range of HP levels in individual hairs, but the general trend suggests that mismatches (homoplasmic variant 1 in buccal and homoplasmic variant 2 in hair) are unlikely. As a rule of thumb: buccal HP sites with levels > 15% are mostly reproduced in hairs (in about 90% of the hairs), while the HP levels can vary. Buccal HP levels < 15% tend to vary substantially in hair HP levels and are often absent in hairs. For a C-stretch-related position, this may appear slightly more frequent which may be due to sequencing bias that affects the detected HP level.

## Figures and Tables

**Table 1:** Observed number of HP events in 26 buccal references categorised by HP level.

Range Minor HP Contribution (as Typed by MPS)	Total Events ^1^	From Which:		
		LHP at Position 16,193	PHP at C-Stretch-Related Sites	PHP Events at Other Sites
3–5%	24	1	19	4
5–10%	19	3	9	7
10–20%	9	5	1	3
20–50%	8	4	0	4
Average number of sites/individual	2.3	0.5	1.1	0.7

^1^ Mixed sites of positions 538, 545 and 550 were not counted since they are suspected sequencing errors (discussed in Section 3.5).

## Supplementary Materials

The following are available online at https://www.mdpi.com/2073-4425/11/11/1355/s1. Table S1: Haplotypes and analysed ranges of donors analysed in this study, Table S2: Proportion of hairs in which buccal HP sites were observed at different levels (by MPS), Figure S1: Illustration of the three scenarios that can arise when comparing data of methods with different detection thresholds, Figure S2: Sanger sequence surrounding position 16,320 for sample X1, Figure S3: HP levels of hairs for HP events also observed in buccal references. Figure S4: Alignment of the mixed sites (suspected errors) observed within the same fragment that contains a C-stretch in HV3.
