# Supplementary material for: Reducing the Number of Mismatches between Hairs and Buccal References When Analysing mtDNA Heteroplasmic Variation by Massively Parallel Sequencing
Source: Genes (Basel). 2020 Nov 16;11(11):1355. doi: 10.3390/genes11111355 (PMC7696041; doi:10.3390/genes11111355)
Supplement: Supplementary file 1 [file genes-11-01355-s001.zip › Supplementary Figures_v2.docx]

|  |  |  |
| --- | --- | --- |

**Supplementary Figure S1**, Illustration of the three scenarios that can arise when comparing data of methods with different detection thresholds. For scenario A, both methods yield the same result. For scenario B, the minor is only called by MPS but is below the detection threshold of Sanger. For scenario C, the minor is below the threshold of both methods and is not called. * The threshold of Sanger in this figure is displayed at 15%, in practice, this threshold is usually somewhere in the range of 10-20%.


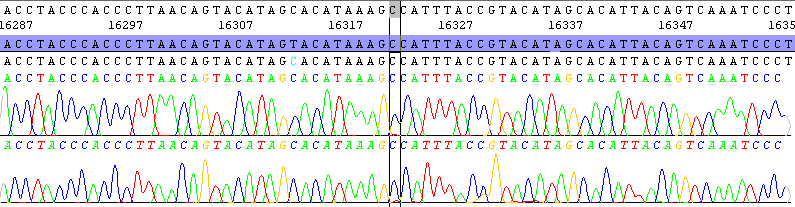


**Supplementary Figure S2**, Sanger sequence surrounding position 16320 for sample X1.

On position 16320 a minimal contribution of T was observed in both orientations. However, the height of the T relative to the C is so low that it most likely wouldn’t be typed as a heteroplasmic position.

**A: B:**

***C:***

**Supplementary Figure S3**, HP levels of hairs for HP events also observed in buccal references (grouped in HP level categories on the x- and Y-axis). The proportion of hairs (total number of investigated occurrences in hairs is indicated as n on the x-axis) in various PHP level categories (see colour coding) is displayed. Data is displayed specifically for PHP position 310 separately (A), PHP positions that are not within a C-stretch (B) and for LHP of position 16193.n (C).

*
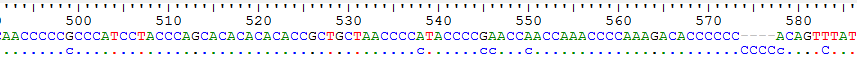
*

**Supplementary Figure S4**, Alignment of the mixed sites (suspected errors) observed within the same fragment that contains a C-stretch in HV3.
